# Sexual and physical abuse and depressive symptoms in the UK Biobank

**DOI:** 10.1186/s12888-021-03207-0

**Published:** 2021-05-11

**Authors:** Anna B. Chaplin, Peter B. Jones, Golam M. Khandaker

**Affiliations:** 1 Department of Psychiatry, University of Cambridge, Cambridge, UK; 2 Cambridgeshire and Peterborough NHS Foundation Trust, Cambridge, UK; 3 MRC Integrative Epidemiology Unit, Population Health Sciences, Bristol Medical School, University of Bristol, Bristol, UK; 4 Centre for Academic Mental Health, Population Health Sciences, Bristol Medical School, University of Bristol, Bristol, UK; 5 Avon and Wiltshire Mental Health Partnership NHS Trust, Bristol, UK

**Keywords:** UK biobank, Observational study, Depressive symptoms, Sexual abuse, Physical abuse

## Abstract

**Background:**

The association between sexual and physical abuse and subsequent depression is well-established, but the associations with specific depressive symptoms and sex differences remain relatively understudied. We investigated the associations of sexual and physical abuse with depressive symptoms in men and women in a large population cohort.

**Methods:**

Observational study based on 151,396 UK Biobank participants. Exposures included self-reported experiences of childhood physical abuse and sexual abuse. Mid-life outcomes included current depressive symptoms score, individual depressive symptoms, and lifetime depression. We used logistic regression to test associations of childhood sexual/physical abuse with depressive outcomes.

**Results:**

Recalled childhood sexual and physical abuse were both associated with current depressive symptoms score in adults. Results for individual symptoms-based analyses suggest that sexual and physical abuse are associated with all depressive symptoms, particularly suicidal behaviours. The associations between lifetime depression and sexual/physical abuse were not fully explained by current depressive symptoms score, indicating that these findings may not be fully attributable to recall bias. There was no indication of differential risk for specific depressive symptoms among men and women.

**Conclusions:**

Sexual and physical abuse are robust risk factors for depression/depressive symptoms regardless of sex. Higher risk of suicidal behaviours associated with childhood sexual/physical abuse are of particular concern. Longitudinal research into sex-specific associations for individual depressive symptoms is required.

**Supplementary Information:**

The online version contains supplementary material available at 10.1186/s12888-021-03207-0.

## Background

Early-life experiences can have long-term effects on health across the lifespan [1]. Sexual abuse and physical abuse are strongly associated with depression [2–5]. These types of abuse are highly prevalent [6, 7] and are associated with depression onset, suboptimal treatment response and poor prognosis [8–12]. Current depressive symptoms may magnify or otherwise alter the recall of early events [13]. However, retrospective self-reporting of abuse by adults has been found to be reliable [14]. Sexual abuse and physical abuse are not reported more often during a depressive episode [14]. Despite extensive literature documenting the association between childhood sexual/physical abuse with adult depression, there are relatively few studies on effects on individual depressive symptoms and potential sex differences.

Previous studies have reported association between abuse and suicidal behaviours in individuals with or without depression [15–18]. Meta-analyses of adults and young people report that sexual abuse and physical abuse are associated with two- to three-fold increased risk for suicide attempts [19, 20]. There are fewer studies of associations between sexual/physical abuse and other depressive symptoms. One study of homeless women suggested that sexual and physical abuse are associated with low self-esteem [21]. Evidence also suggests that sexual and physical abuse are both associated with increased appetite, weight gain, and hypersomnia in adults [22]. Studies of individual depressive symptoms are lacking. Studying symptom-level associations is important because depression is a phenotypically heterogenous condition. Symptom-level associations may provide clues to mechanism of effect.

Depression is more common in women than men [23] but the role of biological sex in the association between sexual/physical abuse and depression is unclear. It is possible that sex may have no mechanistic role beyond higher rates of victimisation among females [24]. On the other hand, it is possible that sex-specific physiological responses to sexual/physical abuse may influence risk for mental health disorders among adults [25]. For example, stress-response pathways may not be the same in both sexes such that the effects of immune activation may be greater in women than men [26]. Revealing a common pattern of sex-specific adversity-related symptoms may be clinically beneficial when considering the most appropriate treatment option for depression. We know of no population-based studies examining associations between sexual/physical abuse and individual depressive symptoms in men and women. While a number of studies from the UK Biobank have examined the association between childhood adversity and depression [27–31], no studies from this sample have investigated the effects of childhood sexual/physical abuse on individual depressive symptoms, and potential sex differences for these associations.

Using the UK Biobank, we explored associations of childhood sexual and physical abuse with individual depressive symptoms in adults. In particular, we tested (i) the strength of associations before and after adjusting for potential covariates; (ii) the consistency and potential sex differences in these associations by assessing the effect of sexual and physical abuse in both men and women; and (iii) we attempted to minimise the possible impact of recall bias by controlling for current depressive symptoms in associations between sexual/physical abuse and lifetime depression.

## Methods

### Description of cohort and sample

The UK Biobank is a long-term UK-based study comprising over 500,000 participants aged between 40 and 69 at baseline, recruited from 22 assessment centres across the United Kingdom between 2006 and 2010 [32].

Informed consent was obtained from participants at recruitment. The UK Biobank contains results of clinical examinations, assays of biological samples, self-reported health behaviour, genome-wide genotyping, and is supplemented by linkage with electronic health records. Details of the UK Biobank resource can be found at www.ukbiobank.ac.uk. UK Biobank received ethical approval from the National Health Service National Research Ethics Service (reference 11/NW/0382). The current analyses were conducted under approved UK Biobank project no. 26999.

**Fig. 1:**
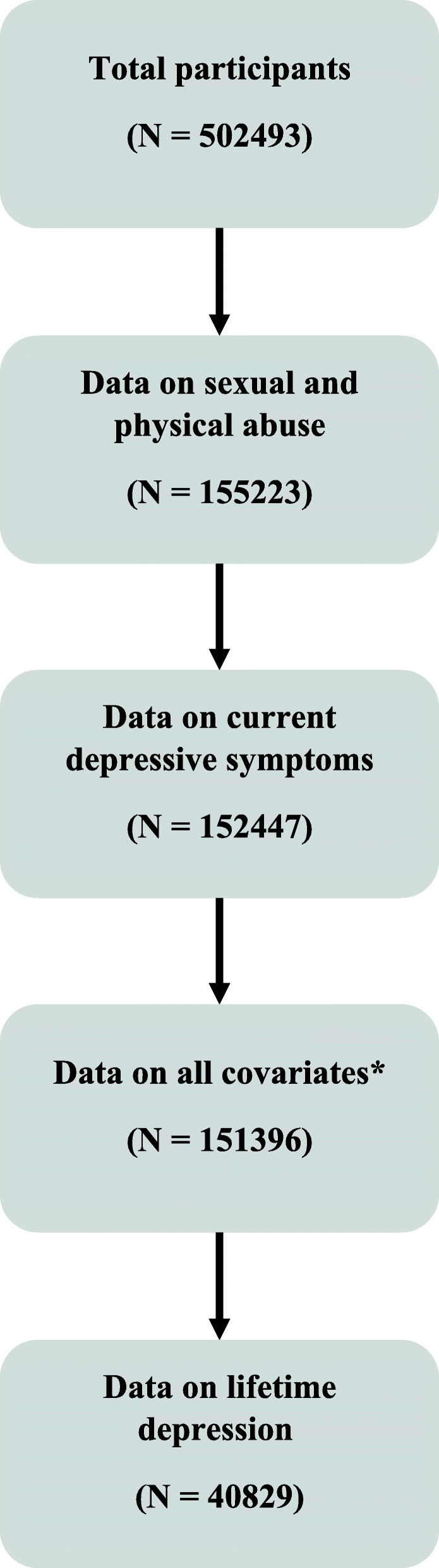
Flow chart showing inclusion of study participants. *Covariates: sex, age, ethnicity, Townsend deprivation index (proxy for socioeconomic status), body mass index, and childhood sexual/physical abuse

The risk set for our analysis comprised 155,223 unrelated participants who answered questions regarding childhood sexual and physical abuse in 2016–2017 (Fig. 1). Of these individuals, 152,447 had data on current depressive symptoms and 151,396 participants had complete data for exposure, outcome, and covariates. The complete cases formed the basis of our main analysis.

### Assessment of childhood sexual and physical abuse

Childhood sexual and physical abuse were assessed in 2016–2017 using an online mental health questionnaire which included information on childhood adversity. Childhood experiences were assessed using the Childhood Trauma Screener [33]. The questionnaire consists of five-point Likert scale items: zero (never true), one (rarely true), two (sometimes true), three (often true), and four (very often true). We used threshold values derived from the validation study [33] to define presence/absence of sexual and physical abuse. Sexual abuse refers to being sexually molested by someone and was defined as: 0 = never true; and 1 = rarely/sometimes/often/very often true. Physical abuse refers to being hit so hard by a family member that it leaves bruises or marks and was defined as 0 = never/rarely true; and 1 = sometimes/often/very often true.

### Assessment of current depressive symptoms in adulthood

Depressive symptoms over the past two weeks were self-assessed in 2016–2017 using the Patient Health Questionnaire (PHQ)-9 as part of the online mental health questionnaire. PHQ-9 scores the nine DSM-IV criteria for depressive symptoms (anhedonia, psychomotor change, change in appetite/weight, fatigue, sleep disturbance, low mood, concentration difficulties, low self-esteem, suicidal behaviours) as zero (not at all), one (several days), two (more than half the days) or three (nearly every day) [34]. A binary variable was created for each of the nine symptoms (0 = not at all/several days, 1 = more than half the days/nearly every day) [34].

We summed the nine individual items to create a total score (0–27) and used a cut-off score of 10 or more to indicate current depressive symptoms [34, 35]. A meta-analysis of over 17,000 participants found that the combined sensitivity (88%; 95% CI = 83–92%) and specificity (85%; 95% CI = 82–88%) of PHQ-9 for detecting major depression was maximised at a cut-off score of 10 or above [36]. Current depressive symptoms score was used as the outcome measure in our analysis.

### Assessment of lifetime depression

We defined adult lifetime depression as self-reported probable moderate and severe major depression, as previously used in UK Biobank [37]. Binary measures of probable moderate and severe major depression were derived from a touchscreen questionnaire assessed at UK Biobank Assessment Centres at baseline [37]. Table 1 demonstrates how (i) a single episode of probable major depression, (ii) probable moderate major depression, and (iii) probable recurrent severe major depression were defined [37]. We used lifetime depression as an alternative depression phenotype (with depressive symptoms score as a control).

**Table 1:** Derivation of depression variables [37]

Criteria	Depression variable	Definition based on criteria
1. Ever felt depressed for a whole week 2. Ever disinterested or unenthusiastic for a whole week 3. Only 1 episode 4. ≥ 2 episodes 5. Episode lasted ≥2 weeks 6. Ever seen a GP for nerves, anxiety, tension or depression 7. Ever seen a psychiatrist for nerves, anxiety, tension or depression	Single episode of probable major depression	{(1) AND (3) AND (5) AND [(6) OR (7)]} **OR** {(2) AND (3) AND (5) AND [(6) OR (7)]}
	Probable recurrent major depression (moderate)	[(1) OR (2)] AND (4) AND (5) AND (6)
	Probable recurrent major depression (severe)	[(1) OR (2)] AND (4) AND (5) AND (7)

### Assessment of covariates

We adjusted for the following covariates: sex, age, ethnicity, Townsend deprivation index (TDI), BMI, and childhood sexual or physical abuse. All covariates were recorded at recruitment (2006–2010), except childhood sexual/physical abuse which were retrospectively reported in 2016–2017.

Sex (female or male) was acquired from NHS records but updated by participants in some cases. Age was based on self-reported date of birth at recruitment. Age (years) was calculated by subtracting year of birth from 2017, i.e. the last year in which online mental health questionnaires were conducted. BMI was calculated as body weight in kilograms divided by height in meters squared. BMI measurements at recruitment (2006–2010) and in 2016–2017 were strongly correlated (beta = 0.94; SE = < 0.01; p = < 0.001). Age and BMI were used as continuous measures.

Ethnicity was originally recorded as White, Mixed, Asian/Asian British, Black/Black British, Chinese, and Other. Given the relatively homogenous nature of the sample, we used ethnicity as a binary variable (White or any other ethnicity).

We included TDI as a proxy for socioeconomic status. TDI is a continuous measure of material deprivation which incorporates unemployment, non-car ownership, non-home ownership, and household overcrowding [38]. These variables are measured in a given area, combined and standardised to produce an overall score for that area. Higher scores represent greater deprivation.

### Statistical analysis

All analyses were carried out using R version 3.6.1. Regression models were estimated before and after adjusting for potential covariates. We examined potential sex differences by: (i) stratifying the sample by sex, and (ii) adjusting regression models for sex.

#### Associations with individual depressive symptoms and PHQ-9 depressive symptoms score

The PHQ-9 current depressive symptoms score distribution was skewed (skew = 2.4; kurtosis = 7.4) and could not be normalised via transformation. We therefore used current depressive symptoms as a measure using a cut-off score of 10 [34–36] .

Logistic regression was used to calculate odds ratios (OR) with 95% confidence intervals (CI) for individual depressive symptoms and current depressive symptoms score. ORs represent the odds of the depressive symptoms measure for individuals exposed to abuse compared with unexposed. Holm-Bonferroni *P*-value correction was performed to correct for multiple testing [39].

To test for interaction with sex, we ran adjusted logistic regression with current depressive symptoms score as the outcome and included interaction terms with sex for all of the covariates and exposures listed in Table 2.

**Table 2:** Characteristics of UK Biobank participants using maximum available sample size

Characteristic	All participants (*N* = 155,223)	Women (*N* = 87,490)	Men (*N* = 67,733)	Difference between sexes (T-test *p*-value)
**Covariates**^a^				
Age (years) – *mean (SD*)	64.8 (7.7)	64.3 (7.7)	65.4 (7.8)	< 0.001
Ethnicity – *no. White (%)*	150,295 (97.2)	84,750 (97.1)	65,545 (97.2)	0.40
Townsend deprivation index – *mean (SD)*	−1.7 (2.8)	− 1.7 (2.8)	− 1.8 (2.8)	< 0.001
Body mass index (kg/m^2^) – *mean (SD)*	26.8 (4.6)	26.3 (4.9)	27.3 (4.0)	< 0.001
Lifetime depression – *no. (%)*	8503 (20.3)	5702 (24.5)	2801 (15.1)	< 0.001
**Exposure**^b^				
Childhood sexual abuse – *no. (%)*	13,612 (8.8)	9673 (11.1)	3939 (5.8)	< 0.001
Childhood physical abuse – *no. (%)*	12,573 (8.1)	6964 (8.0)	5609 (8.3)	0.02
Both types of abuse – *no. (%)*	2678 (1.8)	1946 (2.3)	732 (1.1)	< 0.001
**Outcome**^b^				
*Current depressive symptoms score*				
PHQ-9 0 to 27 – *mean (SD)*	2.7 (3.7)	3.0 (3.8)	2.4 (3.5)	< 0.001
PHQ-9 ≥ 10 – *no. (%)*	8627 (5.7)	5454 (6.4)	3173 (4.8)	< 0.001
Sleep disturbance – *no. (%)*	23,364 (15.1)	14,960 (17.1)	8404 (12.4)	< 0.001
Fatigue – *no. (%)*	17,270 (11.1)	10,638 (12.2)	6632 (9.8)	< 0.001
Change in appetite/weight – *no. (%)*	8225 (5.3)	5645 (6.5)	2580 (3.8)	< 0.001
Low self-esteem – *no. (%)*	6616 (4.3)	4156 (4.8)	2460 (3.6)	< 0.001
Anhedonia – *no. (%)*	6074 (3.9)	3445 (3.9)	2629 (3.9)	0.54
Concentration difficulties – *no. (%)*	5803 (3.7)	3343 (3.8)	2460 (3.6)	0.05
Low mood – *no. (%)*	5362 (3.5)	3263 (3.7)	2099 (3.1)	< 0.001
Psychomotor changes – *no. (%)*	2176 (1.4)	1244 (1.4)	932 (1.4)	0.44
Suicidal behaviours – *no. (%)*	1229 (0.8)	678 (0.8)	551 (0.8)	0.41

^a^Covariates reported at recruitment (2006–2010)

^b^Exposure and outcome measures reported in 2016–2017

#### Sensitivity analysis: association between childhood sexual/physical abuse and adult lifetime depression

To examine possible impact of recall bias, we controlled for current depressive symptoms in logistic regression models testing associations between sexual/physical abuse and lifetime depression. In this way we considered whether reported abuse was associated with later depression and whether this was altered by considering current mood i.e. depressive symptoms score.

## Results

### Characteristics of the sample

The total sample used for analysis (*N* = 155,223) predominantly comprised individuals of White ethnicity (97.2%) and low deprivation (TDI mean = − 1.7; SD = 2.8) (Table 2). Both childhood sexual and physical abuse were experienced by 1.8% of the sample. Sexual abuse was almost twice as common in women (11.1%) than men (5.8%). Physical abuse was more common in men (8.3%) than women (8.0%). Lifetime depression was more common in women (24.5%) than in men (15.1%). Current depressive symptoms score was also higher in women (mean = 3.0; SD = 3.8) than men (mean = 2.4; SD = 3.5).

There was evidence of interaction with sex for TDI (*p* < 0.01) and BMI (*p* = 0.01). There was little evidence of interaction with sex for other variables (age: *p* = 0.74; ethnicity: *p* = 0.58; physical abuse: *p* = 0.41; sexual abuse: *p* = 0.08).

### Association between childhood sexual/physical abuse and adult current depressive symptoms score

In the total sample (*N* = 151,396), sexual abuse was associated with increased risk for current depressive symptoms score after adjusting for potential covariates (adjusted OR = 1.74; 95% CI = 1.63, 1.85) (Table 3). In sex-stratified analysis, sexual abuse was associated with current depressive symptoms score in both men and women (Table 3). ORs were similar between the sexes.

**Table 3:** Odds ratio (95% CI) for the association between childhood sexual abuse and adult depressive outcomes

Outcome^a^	All participants (*N* = 151,396)		Women (*N* = 85,239)		Men (*N* = 66,157)	
	Unadjusted	Adjusted^b^	Unadjusted	Adjusted^b^	Unadjusted	Adjusted^b^
Current depressive symptoms score (PHQ-9 ≥ 10)	2.30 (2.17, 2.44)	1.74 (1.63, 1.85)	2.19 (2.04, 2.35)	1.69 (1.56, 1.81)	2.29 (2.04, 2.56)	1.91 (1.70, 2.15)
*Individual depressive symptoms*						
Sleep disturbance	1.64 (1.57, 1.72)	1.37 (1.31, 1.44)	1.53 (1.45, 1.61)	1.33 (1.26, 1.41)	1.65 (1.52, 1.80)	1.49 (1.36, 1.63)
Fatigue	1.81 (1.72, 1.90)	1.45 (1.38, 1.52)	1.76 (1.66, 1.87)	1.44 (1.36, 1.53)	1.70 (1.55, 1.87)	1.48 (1.34, 1.62)
Change in appetite/weight	2.20 (2.07, 2.35)	1.60 (1.49, 1.71)	2.02 (1.88, 2.17)	1.55 (1.44, 1.68)	2.12 (1.86, 2.41)	1.78 (1.55, 2.04)
Low self-esteem	2.31 (2.16, 2.48)	1.76 (1.64, 1.89)	2.18 (2.01, 2.37)	1.70 (1.56, 1.85)	2.35 (2.06, 2.68)	1.97 (1.72, 2.25)
Anhedonia	1.95 (1.81, 2.10)	1.53 (1.42, 1.66)	1.96 (1.79, 2.15)	1.51 (1.37, 1.66)	1.96 (1.72, 2.24)	1.63 (1.42, 1.87)
Concentration difficulties	2.27 (2.11, 2.45)	1.80 (1.67, 1.94)	2.34 (2.14, 2.55)	1.81 (1.65, 1.99)	2.14 (1.87, 2.44)	1.78 (1.55, 2.04)
Low mood	2.11 (1.95, 2.28)	1.63 (1.50, 1.77)	2.07 (1.89, 2.27)	1.65 (1.50, 1.81)	2.02 (1.75, 2.34)	1.65 (1.42, 1.92)
Psychomotor change	2.37 (2.11, 2.66)	1.78 (1.58, 2.01)	2.42 (2.10, 2.78)	1.75 (1.51, 2.02)	2.29 (1.86, 2.82)	1.84 (1.49, 2.28)
Suicidal behaviours	3.08 (2.69, 3.54)	2.20 (1.90, 2.54)	3.09 (2.60, 3.67)	2.08 (1.73, 2.50)	3.30 (2.62, 4.15)	2.46 (1.94, 3.12)

^a^Individual depressive symptoms defined as experiencing symptom more than half the days/nearly every day

^b^Adjusted for sex (if appropriate), age, ethnicity, Townsend deprivation index, body mass index, and childhood physical abuse

Physical abuse was also associated with current depressive symptoms score in adulthood after adjusting for potential covariates (adjusted OR = 2.17; 95% CI = 2.04, 2.31) (Table 4). Physical abuse as a child was associated with current depressive symptoms score in men and women (Table 4). ORs were similar between the sexes.

**Table 4:** Odds ratio (95% CI) for the association between childhood physical abuse and adult depressive outcomes

Outcome^a^	All participants (*N* = 151,396)		Women (*N* = 85,239)		Men (*N* = 66,157)	
	Unadjusted	Adjusted^b^	Unadjusted	Adjusted^b^	Unadjusted	Adjusted^b^
Current depressive symptoms score (PHQ-9 ≥ 10)	2.86 (2.70, 3.03)	2.17 (2.04, 2.31)	2.86 (2.65, 3.08)	2.12 (1.96, 2.30)	2.91 (2.65, 3.19)	2.24 (2.03, 2.47)
*Individual depressive symptoms*						
Sleep disturbance	1.92 (1.84, 2.01)	1.71 (1.63, 1.79)	1.92 (1.81, 2.03)	1.68 (1.58, 1.78)	1.97 (1.84, 2.12)	1.75 (1.63, 1.88)
Fatigue	2.25 (2.15, 2.36)	1.84 (1.75, 1.94)	2.27 (2.13, 2.41)	1.81 (1.69, 1.93)	2.25 (2.09, 2.43)	1.88 (1.74, 2.03)
Change in appetite/weight	2.61 (2.45, 2.77)	1.93 (1.81, 2.06)	2.60 (2.41, 2.81)	1.89 (1.74, 2.06)	2.69 (2.42, 3.00)	1.98 (1.77, 2.22)
Low self-esteem	2.76 (2.58, 2.96)	2.09 (1.95, 2.24)	2.76 (2.54, 3.01)	2.06 (1.88, 2.25)	2.79 (2.50, 3.11)	2.13 (1.91, 2.39)
Anhedonia	2.57 (2.39, 2.76)	1.94 (1.80, 2.09)	2.58 (2.35, 2.84)	1.92 (1.74, 2.13)	2.55 (2.29, 2.84)	1.96 (1.75, 2.19)
Concentration difficulties	2.72 (2.53, 2.92)	2.05 (1.90, 2.21)	2.75 (2.50, 3.02)	1.98 (1.79, 2.19)	2.68 (2.40, 2.99)	2.12 (1.90, 2.38)
Low mood	2.49 (2.31, 2.69)	1.88 (1.74, 2.04)	2.33 (2.11, 2.58)	1.72 (1.54, 1.91)	2.76 (2.45, 3.10)	2.15 (1.90, 2.24)
Psychomotor change	3.22 (2.89, 3.59)	2.42 (2.16, 2.71)	3.43 (2.98, 3.95)	2.48 (2.13, 2.88)	2.97 (2.51, 3.51)	2.33 (1.96, 2.77)
Suicidal behaviours	4.30 (3.78, 4.90)	2.97 (2.59, 3.41)	4.40 (3.69, 5.24)	2.94 (2.44, 3.54)	4.18 (3.45, 5.08)	3.01 (2.46, 3.69)

^a^Individual depressive symptoms defined as experiencing symptom more than half the days/nearly every day

^b^Adjusted for sex (if appropriate), age, ethnicity, Townsend deprivation index, body mass index, and childhood sexual abuse

All associations were robust to Holm-Bonferroni *P*-value correction for multiple testing (Table S1).

### Association between childhood sexual/physical abuse and adult individual depressive symptoms

In the total sample (*N* = 151,396), sexual abuse was associated with all individual depressive symptoms (Table 3). In women, sexual abuse was most strongly associated with suicidal behaviours, concentration difficulties, and psychomotor change, with adjusted ORs ranging from 1.75 to 2.08 for women (Table 3). In men, sexual abuse was most strongly associated with suicidal behaviours, low self-esteem, and psychomotor change, with adjusted ORs ranging from 1.84 to 2.46 (Table 3).

Physical abuse was also associated with all individual depressive symptoms (Table 4). Physical abuse was most strongly associated with suicidal behaviours, psychomotor change, and low self-esteem in women, with adjusted ORs ranging from 2.06 to 2.94. Physical abuse was most strongly associated with change in suicidal behaviours, psychomotor change, and low mood in men, with adjusted ORs ranging from 2.15 to 3.01 (Table 4).

All associations remained after covariate adjustment and Holm-Bonferroni *P*-value correction for multiple testing (Table S1).

### Sensitivity analysis: association between childhood sexual/physical abuse and adult lifetime depression

In the total sample of participants with data on lifetime depression (*N* = 40,829), sexual abuse was associated with lifetime depression (OR = 2.12; 95% CI = 1.97, 2.29) (Table 5). Evidence for this association remained after adjusting for potential covariates including current depressive symptoms score (adjusted OR = 1.58; 95% CI = 1.46, 1.72). In sex-stratified analysis, sexual abuse was associated with lifetime depression in both men and women even after adjusting for current depressive symptoms score. ORs were similar between the sexes.

**Table 5:** Sensitivity analysis: Odds ratio (95% CI) for the association between sexual/physical abuse as a child and lifetime depression in adulthood

Participants	Sample (no.)	Lifetime depression (%)	Childhood exposure	Odds ratio (95% CI) for lifetime depression		
				Unadjusted	Adjusted 1^a^	Adjusted 2^b^
All	40,829	20.3	Sexual abuse	2.12 (1.97, 2.29)	1.77 (1.64, 1.91)	1.58 (1.46, 1.72)
			Physical abuse	2.00 (1.85, 2.16)	1.74 (1.60, 1.89)	1.45 (1.33, 1.58)
Women	22,683	24.5	Sexual abuse	2.00 (1.83, 2.18)	1.79 (1.63, 1.96)	1.61 (1.47, 1.77)
			Physical abuse	2.03 (1.84, 2.25)	1.71 (1.54, 1.90)	1.45 (1.30, 1.62)
Men	18,146	15.1	Sexual abuse	1.90 (1.64, 2.20)	1.74 (1.50, 2.02)	1.52 (1.30, 1.78)
			Physical abuse	2.00 (1.77, 2.28)	1.78 (1.57, 2.03)	1.45 (1.26, 1.66)

^a^Adjusted for sex (if applicable), age, ethnicity, Townsend deprivation index, body mass index, and sexual/physical abuse as appropriate

^b^Additional adjustment for PHQ-9 depressive symptoms score

In the total sample (*N* = 40,829), physical abuse was associated with lifetime depression (OR = 2.00; 95% CI = 1.85, 2.16) (Table 5). Evidence for this association remained after adjusting for potential covariates including current depressive symptoms score (adjusted OR = 1.45; 95% CI = 1.33, 1.58). In sex-stratified analysis, physical abuse was associated with lifetime depression in both men and women even after adjusting for current depressive symptoms score. ORs were similar between the sexes.

## Discussion

The aim of this study was to explore the well-established relationship between sexual and physical abuse and adult depression at the symptom level. To our knowledge, this is one of the first studies to consider the effect of childhood sexual/physical abuse on multiple depressive symptoms in both men and women. Our results indicate three key findings. First, childhood sexual and physical abuse were both associated with all depressive symptoms. Second, childhood sexual and physical abuse were particularly strongly associated with suicidal behaviours. Three, the pattern of association, regarding ORs and effects on depressive symptoms, was similar between the sexes.

Recent UK Biobank studies have considered heterogeneity within the depression syndrome in its association with childhood sexual and physical abuse [27–31]. A cross-sectional study suggested that atypical depression (classed as hypersomnia and weight gain) is more strongly associated with sexual and physical abuse than typical depression [27]. This adversity-related subtype also exhibited sociodemographic differences; atypical depression was associated with female sex, earlier onset of illness, and lower income [27]. Moreover, genetic studies from the UK Biobank indicate that heterogeneity within the depression syndrome is a function of environmental exposures, such as childhood adversity [29, 30]. For example, genetic contribution to depression appears to be greater when childhood adversity is present [30]. We have added to this literature by investigating sexual/physical abuse, individual depressive symptoms and potential sex differences.

Our results confirm that environmental factors, such as childhood sexual/physical abuse, play an important role in the onset of depressive symptoms [40]. We found that both childhood sexual and physical abuse were strongly associated with all depressive symptoms, particularly suicidal behaviours. Consistent with our findings, an observational study of adolescents found that 70% of participants who had experienced sexual abuse and physical abuse also experienced suicidal thoughts [41]. Experiences of childhood sexual/physical abuse may lead to feelings of entrapment, habituation to pain, and reduced fear for death which may result in greater capacity for suicidal behaviours as a means of escape [19]. A recent study has suggested that adverse social relationships during childhood can also contribute to depressive symptoms including suicidal behaviours [42]. For example, a study of 1277 students reported that violent treatment of the mother is associated with almost four-fold increased risk in attempted suicide [43]. Identifying and treating adults and children who have experienced sexual/physical abuse and other forms of adversity may help prevent the onset of depressive symptoms including suicidal behaviours. Adults with depression and history of abuse often do not respond to combined treatment with anti-depressant medication and psychological therapy, but could be given more intensive alternative interventions [12]. Children exposed to abuse could be screened for symptoms and given appropriate support and therapy [44], which may help to decrease the risk of future adverse health consequences.

Childhood sexual and physical abuse may become “biologically embedded” in the body via stress-response pathways [45, 46]. Hypothalamic-pituitary-adrenal (HPA) axis dysregulation, leading to high cortisol reactivity, may contribute to the somatic symptoms of depression. For example, HPA axis dysregulation may result in changes in appetite/weight via emotional eating mechanisms that serve to dispel unwanted distress [47]. The psychological consequences of exposure to sexual/physical abuse may lead to HPA axis dysregulation, inflammation, and subsequent psychological depressive symptoms [45–47]. Inflammation is implicated in depression [48, 49], and may be particularly relevant in the context of childhood adversity [50, 51]. For example, reward sensitivity, a process facilitated by inflammatory cytokines, is blunted by sexual and physical abuse [46, 52]. These types of abuse have also been associated with blunted cortisol in children as well as alteration to immune and inflammatory responses and increased risk for infection [53]. Longitudinal studies that consider the temporality of sexual/physical abuse, HPA axis dysregulation, inflammation, and depressive symptoms are required.

We found no evidence that the association between childhood sexual/physical abuse and adult depressive symptoms differs between men and women. This is consistent with the idea that biological sex has no mechanistic role other than higher rates of victimisation among females [24]. Indeed, in our sample childhood sexual abuse was almost twice as common in women than in men. Depression has also been reported to affect women twice as often as men [54]. Consistently, over 50% more women in our sample had lifetime depression than men. A greater percentage of women in our sample also experienced sleep disturbance, fatigue, change in appetite/weight, low self-esteem, and low mood. A recent study suggested that this disparity in risk for depression between men and women peaks in adolescence [55], although it is unclear how much childhood sexual/physical abuse contributes to this disparity. A study of adolescents reported that females with a history of sexual/physical abuse were more depressed than males with a history of sexual/physical abuse [41]. Conversely, we found that odds ratios for associations between sexual/physical abuse and depression were generally larger (although not significantly) for men than women. It is possible that covariates explained a greater amount of the association between childhood sexual/physical abuse and depression in women than in men. Alternatively, this finding might be a type one error.

A key limitation of this study is its cross-sectional design. A longitudinal approach would have been helpful to better understand trajectories from childhood sexual/physical abuse to depressive symptoms in adulthood. The nature of UK Biobank data collection meant this was not possible. Furthermore, the use of self-reported recall of childhood sexual/physical abuse by adults could introduce some level of recall bias. We attempted to minimise the impact of recall bias by adjusting for current mood. We found that differential recall of negative events by depressed individuals was not the sole explanation for the association between sexual/physical abuse and depressive symptoms, but other factors could still contribute to bias. Another limitation is that we did not take into account the duration, frequency or severity of abuse; longer periods of abuse may have a more profound effect on specific depressive symptoms than single events. In addition, the depressive symptoms assessed could be part of medical or psychiatric conditions other than depression. Finally, residual confounding may still explain the association between sexual/physical abuse and subsequent depressive symptoms.

Childhood sexual and physical abuse are both robust risk factors for all depressive symptoms, regardless of sex. Suicidal behaviours was particularly strongly associated with these types of abuse. Longitudinal studies with large sample sizes are required to investigate biopsychosocial mechanisms affecting the relationship between childhood sexual/physical abuse and depressive symptoms in men and women.

## Supplementary Information

**Additional file 1 Table S1.** Corrected *P*-values for association between childhood sexual/physical abuse and adult depressive outcomes using the Holm-Bonferroni Method.

## Data Availability

The dataset supporting the conclusions of this article is available on the UK Biobank website, www.ukbiobank.ac.uk.

## Abbreviations

BMI: body mass index
TDI: Townsend deprivation index
PHQ-9: Patient Health Questionnaire Nine
OR: odds ratio
CI: confidence interval
DSM-IV: Diagnostic and Statistical Manual of Mental Disorders fourth edition
HPA: hypothalamic–pituitary–adrenal
SD: standard deviation
