# Supplementary material for: Sexual and physical abuse and depressive symptoms in the UK Biobank
Source: BMC Psychiatry. 2021 May 11;21:248. doi: 10.1186/s12888-021-03207-0 (PMC8127207; doi:10.1186/s12888-021-03207-0)
Supplement: Supplementary file 1 — Additional file 1 Table S1. Corrected P-values for association between childhood sexual/physical abuse and adult depressive outcomes using the Holm-Bonferroni Method. [file 12888_2021_3207_MOESM1_ESM.docx]

| **Table S1**  **Corrected *P*-values for association between childhood sexual/physical abuse and adult depressive outcomes using the Holm-Bonferroni Method** | | | | | | |
| --- | --- | --- | --- | --- | --- | --- |
| **Depressive symptom*** | **Original *P*-value‡** | | | **Corrected *P*-value** | | |
|  | **All participants** | **Women** | **Men** | **All participants** | **Women** | **Men** |
| **Sexual abuse** | | | | | | |
| Current depressive symptoms | <0.001 | <0.001 | <0.001 | <0.001 | <0.001 | <0.001 |
| Sleep disturbance | <0.001 | <0.001 | <0.001 | <0.001 | <0.001 | <0.001 |
| Fatigue | <0.001 | <0.001 | <0.001 | <0.001 | <0.001 | <0.001 |
| Change in appetite/weight | <0.001 | <0.001 | <0.001 | <0.001 | <0.001 | <0.001 |
| Low self-esteem | <0.001 | <0.001 | <0.001 | <0.001 | <0.001 | <0.001 |
| Anhedonia | <0.001 | <0.001 | <0.001 | <0.001 | <0.001 | <0.001 |
| Concentration difficulties | <0.001 | <0.001 | <0.001 | <0.001 | <0.001 | <0.001 |
| Low mood | <0.001 | <0.001 | <0.001 | <0.001 | <0.001 | <0.001 |
| Psychomotor change | <0.001 | <0.001 | <0.001 | <0.001 | <0.001 | <0.001 |
| Suicidal behaviours | <0.001 | <0.001 | <0.001 | <0.001 | <0.001 | <0.001 |
| **Physical abuse** | | | | | | |
| Current depressive symptoms | <0.001 | <0.001 | <0.001 | <0.001 | <0.001 | <0.001 |
| Sleep disturbance | <0.001 | <0.001 | <0.001 | <0.001 | <0.001 | <0.001 |
| Fatigue | <0.001 | <0.001 | <0.001 | <0.001 | <0.001 | <0.001 |
| Change in appetite/weight | <0.001 | <0.001 | <0.001 | <0.001 | <0.001 | <0.001 |
| Low self-esteem | <0.001 | <0.001 | <0.001 | <0.001 | <0.001 | <0.001 |
| Anhedonia | <0.001 | <0.001 | <0.001 | <0.001 | <0.001 | <0.001 |
| Concentration difficulties | <0.001 | <0.001 | <0.001 | <0.001 | <0.001 | <0.001 |
| Low mood | <0.001 | <0.001 | <0.001 | <0.001 | <0.001 | <0.001 |
| Psychomotor change | <0.001 | <0.001 | <0.001 | <0.001 | <0.001 | <0.001 |
| Suicidal behaviours | <0.001 | <0.001 | <0.001 | <0.001 | <0.001 | <0.001 |
| * Current depressive symptoms: PHQ-9 score ≥ 10. Individual depressive symptoms defined as experiencing symptom more than half the days/nearly every day.  ‡ *P*-values correspond to adjusted odds ratios (ORs) presented in Table 2 & 3: ORs adjusted for sex (if applicable), age, ethnicity, Townsend deprivation index, body mass index, and sexual/physical abuse as appropriate. | | | | | | |
